# From chromatin to crop: epigenetic innovations in bioenergy systems

**DOI:** 10.3389/fpls.2025.1687164

**Published:** 2026-02-02

**Authors:** Saddie Vela, Christina R. Steadman

**Affiliations:** Genomics & Bioanalytics Group, Biosciences Division, Los Alamos National Laboratory, Los Alamos, NM, United States

**Keywords:** energy crops, epigenetics, crop improvement, bioenergy, crop resilience, CRISPR

## Abstract

Energy crops encompass a diverse array of plant species cultivated primarily as a source of biomass for energy generation and biofuel production. As such, they play a pivotal role in the transition to sustainable energy systems. However, their productivity is often limited by environmental stresses, nutrient availability, and the need for optimized yield. While traditional breeding and genetic engineering have driven improvements, challenges such as narrow genetic diversity, long development cycles, trait instability, and unexpected gene interactions remain. Epigenetics offers a largely untapped opportunity to overcome these constraints by regulating gene expression through mechanisms that are dynamic, finely tuned, and responsive to environmental and developmental cues. Epigenetic modifications including DNA methylation, histone post-translational changes, and small non-coding RNAs influence nearly all aspects of plant development and physiology, including traits central to bioenergy crops. While these mechanisms are well characterized in model species such as *Arabidopsis thaliana*, they remain underexplored in many purpose-grown energy crops. This review summarizes the current state of knowledge of epigenetic regulation in bioenergy species, explores how these mechanisms can be leveraged to enhance crop resilience and productivity, and identifies gaps in our understanding. By characterizing epigenetic mechanisms and harnessing epigenetic variation, we can expand the toolkit for developing resilient, high-yielding bioenergy crops to meet future environmental and energy demands.

## 1 Introduction

Rising energy demand driven by population growth and industrialization exacerbates pollution, depletes fossil fuels, and creates electricity shortages (Wang et al., 2023). These challenges have made the global energy transition a pressing priority, requiring sustainable alternatives that reduce carbon emissions, preserve ecosystems, and protect food security. Energy derived from biomass rather than fossil fuels, termed *bioenergy*, is generated from organic materials such as plants, agricultural residues, and waste (Henrich et al., 2015). Biomass currently supplies nearly 10% of the world’s primary energy demand (Errera et al., 2023) but is still primarily derived from traditional sources. Firewood alone accounts for 67% of global bioenergy, while other biomass residues contribute another 23% (Errera et al., 2023). These sources are often inefficient, land-intensive, and environmentally unsustainable, thereby limiting bioenergy’s contribution toward a decarbonized future. To overcome these limitations, attention has shifted toward purpose-grown energy crops—plant species cultivated specifically for energy production. These crops represent a transformative shift from subsistence biomass use to modern, scalable energy systems. By 2050, bioenergy is projected to supply between 7.5% and 37% of global primary energy demand (Errera et al., 2023), driven in large part by advances in crop-based biofuels such as ethanol, biodiesel, and methane. Unlike traditional firewood, purpose-grown energy crops are fast-growing, high-yielding, and capable of producing energy-rich biomass with a favorable carbon balance. During cultivation, they absorb atmospheric CO_2_, helping offset emissions generated during fuel combustion (Lemus and Lal, 2005).

**Figure 1:**
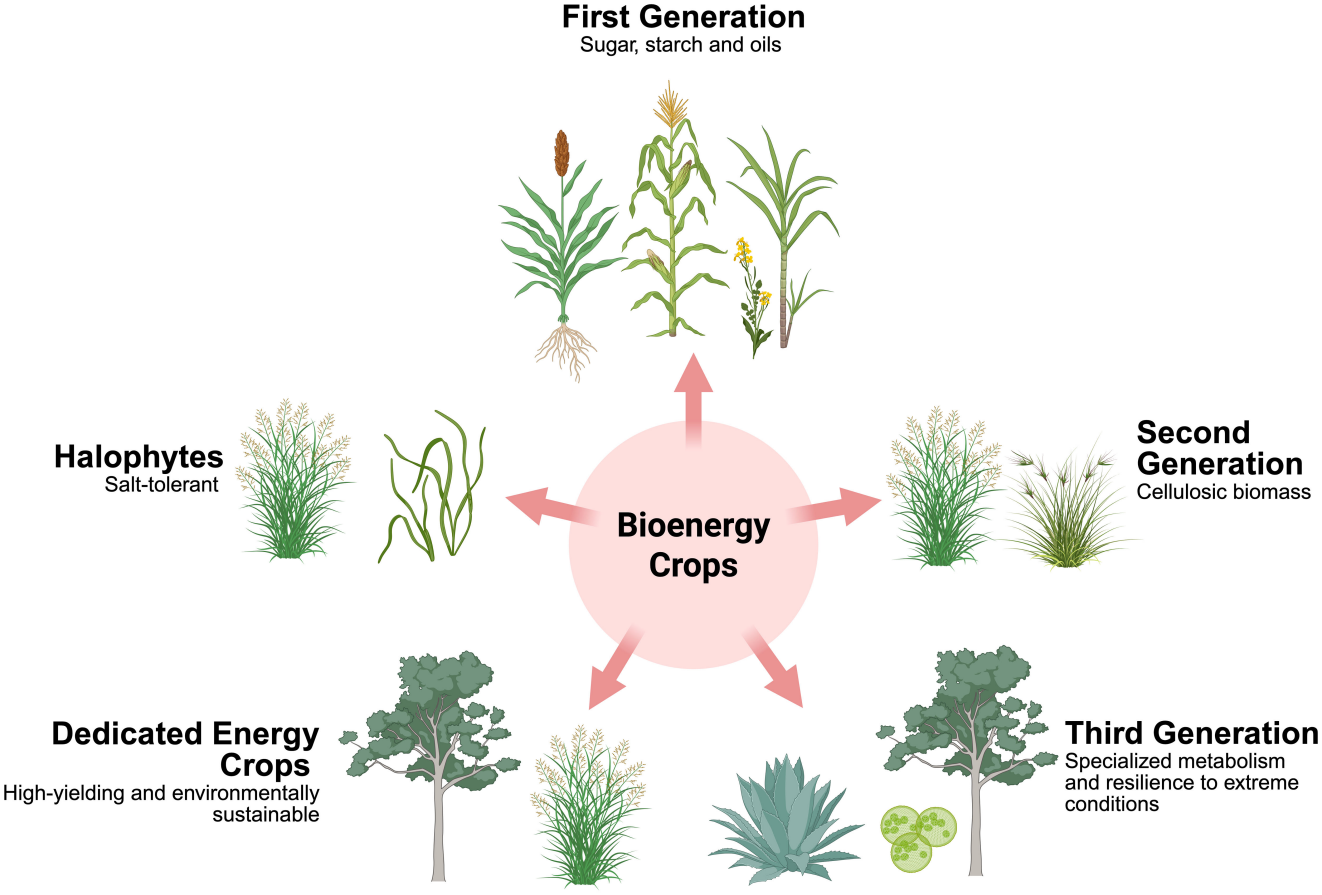
Classification of bioenergy crops depicted with representative plant species, illustrating categories such as first generation, second generation, and third generation bioenergy crops, dedicated energy crops, and halophytes, along with their distinctive physiological traits.

Bioenergy crops can be broadly categorized into first-, second-, and third-generation crops (Kour et al., 2019), along with dedicated energy crops and halophytes, each exhibiting distinct physiological and ecological characteristics (see **Table 1**). First-generation bioenergy crops primarily comprise widely cultivated food plants such as maize (*Zea mays*) and sugarcane (*Saccharum officinarum*), valued for their high starch and sugar content (Yadav et al., 2019). However, their use in biofuel production is constrained by competition with food supply and relatively high production costs (Sadaqat et al., 2025). Second-generation bioenergy crops predominantly include perennial grasses such as switchgrass (*Panicum virgatum*) and miscanthus (*Miscanthus* × *giganteus*), as well as lignocellulosic biomass sources derived from crop residues such as wheat straw and corn stover. These feedstocks offer greater energy efficiency, as they produce more usable energy relative to the energy required for cultivation and processing, while also reducing greenhouse gas (GHG) emissions and lowering environmental impact compared to first-generation crops (Sanderson and Adler, 2008; Oliver et al., 2009). Third-generation bioenergy feedstocks, including microalgae and plants with specialized metabolic pathways such as crassulacean acid metabolism (CAM: a carbon fixation strategy that temporally separates CO_2_ uptake and assimilation to minimize water loss) are characterized by high resource-use efficiency and adaptations to extreme environments, enabling more efficient biomass conversion and resilience under diverse conditions (Debnath and Das, 2022; Wang et al., 2024). Dedicated energy crops, often high-yielding perennials such as short rotation coppice species like willow (*Salix* sp.) and poplar (*Populus* sp.) are cultivated specifically for sustainable biomass production with minimal agronomic inputs (Guldhe et al., 2017; Yadav et al., 2019). Halophytes, such as pickleweed (*Salicornia bigelovii*) and sea-blite (*Suaeda* sp.), are characterized by their tolerance to saline and degraded soils, and contribute significantly to ecosystem rehabilitation, carbon sequestration, and biofuel production (Ventura et al., 2015; Sharma et al., 2016). Halophytes can serve as sources of lignocellulosic biomass or seed oil, with the latter benefiting from high secondary metabolite content that improves biofuel conversion efficiency (Sharma et al., 2016) (**Figure 1**). Fourth-generation bioenergy crops (although outside of the focus for this review) primarily utilize genetically engineered microorganisms, particularly microalgae, to produce biofuels by enhancing their ability to capture CO_2_ and produce lipids or other desired fuel precursors (Kour et al., 2019: Cavelius et al., 2023).

**Table 1:** Classification of bioenergy crops with representative species and their characteristics, indicating their roles in sustainable biofuel production and environmental benefits.

Generation/Type	Crop examples	Main characteristics
First-Generation Bioenergy Crops	Sweet sorghum (*Sorghum bicolor*), Corn (*Zea mays*), Sugarcane (*Saccharum officinarum*), Oil palm (*Elaeis guineensis*), Rapeseed/Canola (*Brassica napus*), Camelina (*Camelina sativa*)	Food crops used for bioethanol or biodiesel; high sugar or oil content; compete with food supply; higher production cost
Second-Generation Bioenergy Crops	Switchgrass (*Panicum virgatum*), Miscanthus (*Miscanthus giganteus*), Alfalfa (*Medicago sativa*), Reed canary grass (*Phalaris arundinacea*), Napier grass (*Pennisetum purpureum*), Bermuda grass (*Cynodon dactylon*)	Perennial grasses; lignocellulosic biomass; not widely cultivated for human consumption; lower input requirements; better GHG balance; marginal land use
Third-Generation Bioenergy Crops	Boreal plants (*Phleum pratense*, *Phalaris arundinacea*), CAM plants (*Agave tequilana*, *Opuntia ficus-indica*), Eucalyptus (*Eucalyptus globulus*), Microalgae (*Chlorella vulgaris*, *Nannochloropsis* sp.)	Advanced biofuels; high photosynthetic efficiency; tolerate extreme environments; edible in some cases but not mass-produced for food; low land use footprint
Dedicated Energy Crops	Poplar (*Populus* sp.), Willow (*Salix* sp.), Birch (*Betula* sp.), Giant reed (*Arundo donax*), Castor bean (*Ricinus communis*), Jatropha (*Jatropha curcas*), Pongamia (*Pongamia pinnata*), Vernicia (*Vernicia fordii*)	Primarily grown for biomass or oil rather than food; short rotation; high biomass yield; minimal processing; environmental co-benefits
Halophytes	*Kosteletzkya pentacarpos*, *Rhizophora* sp., *Tamarix* sp., *Casuarina* sp., *Eucalyptus* sp., *Prosopis* sp., *Melaleuca* sp.*, Suaeda* sp.*, Salicornia bigelovii*, Switchgrass (*Panicum virgatum*)	Salt-tolerant; grow in saline/marginal lands; sequester carbon; phytoremediation of soil; biomass used for biodiesel; edible in some cases but not mass-produced for food

Traditional cultivation of bioenergy crops largely relies on optimal environmental conditions, fertile arable land, and energy-intensive inputs, which limits scalability due to competition with food crops and sustainability concerns (Sadaqat et al., 2025). Next-generation cultivation strategies emphasize growing bioenergy crops on marginal lands using minimal or waste-derived inputs, reducing environmental impacts and land-use conflicts. In industrial agriculture, conventional breeding techniques are slow and limited by species compatibility and genetic diversity, hindering the development of stress-tolerant, resource-efficient varieties for marginal environments. Genetic engineering enables targeted trait improvement but faces challenges, including inconsistent performance across environments due to complex gene by environment interactions. Engineered traits may degrade or be silenced over generations due to genetic mechanisms, raising concerns about trait stability (Dietz-Pfeilstetter, 2010). Additionally, pleiotropic effects, gene flow to wild relatives, and genetic drift can influence the persistence and ecological behavior of introduced genes, potentially leading to unpredictable outcomes or loss of the trait through recombination or selection pressures (Chandler and Dunwell, 2008; Caradus, 2023). The inherent limitations of conventional breeding and genetic engineering emphasize the necessity of exploring alternative mechanisms that provide heritable yet flexible control over phenotypic traits.

Epigenetic processes, such as DNA methylation, histone post-translational modifications, and small non-coding RNAs regulate nearly all aspects of plant development and physiology, including traits important to bioenergy production. These dynamic, reversible, and heritable layers of regulation offer a valuable source of phenotypic variation, enabling more flexible and rapid crop improvement than traditional breeding or genetic engineering alone (Samantara et al., 2021). Notably, many traits selected through breeding have epigenetic underpinnings, indicating the importance of incorporating epigenetic variation into energy crop improvement strategies. Failing to consider epigenetic variation in crop breeding and production can lead to severe yield losses, as exemplified by the mantled phenotype in tissue-cultured African oil palm, a major biofuel crop cultivated for its high oil content. In this case, clonal propagation in African oil palm induces hypomethylation (loss of DNA methylation) at the Karma locus, causing aberrant splicing that disrupts normal reproductive organ development and leads to sterile, seedless fruit with significantly reduced oil yield (Ong-Abdullah et al., 2015). This epigenetic instability demonstrates the need to integrate epigenetic knowledge and tools to avoid detrimental phenotypes and ensure stable, high yields.

Epigenetic marks on specific genes that result in heritable phenotypic variation, known as epialleles, have been associated with important agronomic traits in both wild and domesticated species (reviewed by Catoni and Cortijo, 2018; Varotto et al., 2022), emphasizing the potential of epigenetic variation as a tool for targeted crop trait development. Both heritable and non-heritable epigenetic mechanisms hold considerable promise for enhancing bioenergy crop productivity: stable epialleles can be targeted through breeding for durable trait enhancement, while environmentally responsive, non-heritable modifications provide flexible and reversible gene regulation that can improve crop performance under fluctuating conditions (Tonosaki et al., 2022). Unlike conventional breeding, which relies on genetic variation and multi-generational selection, epigenetic modifications provide a means of rapidly adapting to environmental stimuli and may be utilized to modify traits critical to bioenergy crop efficiency and resilience (Kakoulidou et al., 2021).

This review summarizes the role of epigenetic regulation in enhancing traits important to bioenergy crop productivity and efficiency. Emphasis is placed on traits underlying stress resiliency, cell wall composition, biomass yield, nutrient use efficiency, and oil content, all of which are influenced by epigenetic mechanisms in diverse plant systems. While much of the foundational knowledge derives from model species, such as *Arabidopsis thaliana*, our review considers cross-species conservation of these regulatory pathways and their relevance to established bioenergy crops. Recent advances in epigenomic technologies are discussed for their role in deepening understanding of these mechanisms—ranging from long-read platforms like Oxford Nanopore and PacBio for direct DNA methylation profiling, to single-cell techniques that resolve chromatin accessibility, 3D genome architecture, and methylation at the cellular resolution. We also discuss emerging strategies for epigenome editing using CRISPR/dCas9 systems that enable precise, programmable modulation of chromatin states without altering DNA sequence. By integrating foundational knowledge with cutting-edge tools and technologies, this review provides a forward-looking framework for harnessing epigenetic regulation to accelerate the development of adaptable, high-yielding, and environmentally sustainable bioenergy crops.

## 2 The fundamentals of epigenetics

Epigenetic mechanisms regulate gene expression without altering the underlying DNA sequence through dynamic modifications to DNA, RNAs, histone proteins, and chromatin structure. Collectively, these processes shape the plant epigenome—the genome-wide chromatin landscape—through semi-reversible and often heritable changes (Agarwal et al., 2020). Among these mechanisms, DNA methylation, primarily in the form of 5-methylcytosine (5mC) in CG, CHG, and CHH sequence contexts (where H = A, T, or C), plays diverse roles in transcriptional regulation in plants (Schmitz et al., 2019). Gene body CG methylation is often associated with active transcription, while CG methylation in promoter regions is typically associated with transcriptional repression (Bewick and Schmitz, 2017; Zhang and Zhu, 2025). While plants have variability in epigenetic processes, the most explicitly detailed studies have been done in *Arabidopsis thaliana*; these studies provide our “textbook knowledge” of epigenetics in plants. In *Arabidopsis thaliana*, DNA methylation is maintained by five DNA methyltransferases: METHYLTRANSFERASE1 (MET1), CHROMOMETHYLASE 2 (CMT2), CHROMOMETHYLASE 3 (CMT3), and DOMAINS REARRANGED METHYLTRANSFERASE 1 and 2 (DRM1 and DRM2) each acting on specific sequence contexts (Law and Jacobsen, 2010; Stroud et al., 2013, 2014). The canonical pathway primarily focuses on maintenance methylation during DNA replication and is facilitated by MET1 to maintain CG methylation, often in coordination with VIM proteins and CMT3, which catalyze CHG methylation through a reinforcing feedback loop with histone H3K9 methylation (Law and Jacobsen, 2010; Stroud et al., 2013, 2014). The non-canonical pathway depends on the RNA-directed DNA methylation (RdDM) machinery, where small interfering RNAs (siRNAs) guide *de novo* methylation, particularly targeting transposons and repetitive DNA sequences (Ali and Tang, 2025). This pathway involves the recruitment of methyltransferases such as DRM2 and CMT2, which establish and maintain CHH methylation, the most dynamic methylation context in plants (Matzke and Mosher, 2014). These methylation patterns are counterbalanced by active DNA demethylation pathways, primarily mediated by REPRESSOR OF SILENCING1 (ROS1)-family DNA glycosylases that excise 5-methylcytosine and initiate base excision repair (Zhang et al., 2022). These pathways, along with histone modifications and siRNA-directed processes, enable faithful transmission of epigenetic information and dynamic regulation of gene expression across generations.

The stable propagation of epigenetic information across generations, or transgenerational epigenetic inheritance, is well documented plants, where DNA methylation is the most often used mark for this process (Hollwey et al., 2023). The stability of epigenetic information is primarily due to limited epigenomic resetting in plants and late segregation of the germline from somatic tissue, which together increase the likelihood that environmentally induced epigenetic changes can be inherited by subsequent generations (Bouyer et al., 2017). In *Arabidopsis*, CG methylation is the most stably inherited, CHG methylation persists due to its self-reinforcing loop (Mathieu et al., 2007; Stroud et al., 2013), and CHH methylation, though more variable, can play a role in transgenerational regulation, particularly under environmental stress (Panda et al., 2023).

Non-coding RNAs including microRNAs (miRNAs), small interfering RNAs (siRNAs), and long non-coding RNAs (lncRNAs) also influence gene expression by modulating chromatin states, transcriptional activity, mRNA stability and translational efficiency (Ali and Tang, 2025). In *Arabidopsis thaliana*, miRNAs can directly target transcripts encoding epigenetic regulators, which repress DRM2 and CMT3, crucial for DNA methylation maintenance and *de novo* methylation (Jha and Shankar, 2014). Moreover, 24-nt long miRNAs (miRNAs) can induce RdDM at both cis- and trans- target sites, thereby influencing transcriptional silencing without requiring RNA-dependent RNA polymerase 2 (RDR2) (Jiang, 2020). Secondary siRNAs, including trans-acting siRNAs (ta-siRNAs) and phased siRNAs (phasiRNAs), are generated through miRNA-triggered cleavage of non-coding or coding transcripts and similarly guide RdDM, contributing to locus-specific DNA methylation and transcriptional repression (Yoshikawa et al., 2005; Axtell et al., 2006). LncRNAs further modulate chromatin states by serving as molecular scaffolds that recruit histone-modifying complexes (Heo and Sung, 2011).

Histone modifications, such as acetylation and methylation, further influence gene activity by altering chromatin structure and accessibility. Histone acetylation is dynamically regulated by histone acetyltransferases (HATs), which add acetyl groups to lysine residues to promote gene activation, and histone deacetylases (HDACs), which remove acetyl groups to repress transcription (Kumar et al., 2021). HATs in *Arabidopsis* include 12 enzymes grouped into families such as HAG, HAC, and HAF, and they promote gene activation by neutralizing positive charges on histones, loosening DNA–histone interactions, and increasing chromatin accessibility (Oliva et al., 1990; Earley et al., 2007; Boycheva et al., 2014). There are several classes of HDACs, including class I enzymes like HDAC6, HDAC9, and HDAC19, which regulate gene expression by removing acetyl groups, leading to chromatin condensation and repression. HDAC6 also uniquely silences transposable elements, while HDAC9 and HDAC19 mainly target euchromatic genes (Fong et al., 2006; Yuan et al., 2019; Chen et al., 2020; Hung et al., 2020; Feng et al., 2021; Saharan et al., 2024). Both HATs and HDACs lack inherent DNA-binding specificity and rely on interactions within epigenetic complexes to target specific genomic regions. The balance between HAT and HDAC activities is critical for dynamic gene expression regulation in plants (Boycheva et al., 2014; Chen et al., 2020; Feng et al., 2021; Le et al., 2025).

Additionally, histone variants and chromatin remodeling complexes work together to modulate nucleosome dynamics, which is crucial for transcriptional regulation (Le et al., 2025). In plants, variants of histones H2A, H3, H2B, and H1 increase chromatin diversity and influence gene expression (Foroozani et al., 2022). For example, H2A.X functions in DNA damage response (Lang et al., 2012), H2A.W promotes chromatin condensation in heterochromatin (Yelagandula et al., 2014), and H2A.Z is enriched near transcription start sites and within low-expression gene bodies, thereby influencing transcriptional regulation (Coleman-Derr and Zilberman, 2012). Among H3 variants, centromeric H3 localizes specifically to centromeres, while H3.1 and H3.3 differ in their replication-dependent and independent deposition and associate with repressive and active chromatin states, respectively (Stroud et al., 2012; Lu et al., 2018).

Chromatin remodeling complexes are ATP-dependent enzymes that regulate gene expression by altering histone–DNA interactions to control DNA accessibility. They modify chromatin through nucleosome repositioning, eviction, or incorporation of histone variants, facilitating or restricting access to transcriptional machinery. Major remodeler families include SWI/SNF, ISWI, CHD, INO80, and SWR1, each with distinct domain architectures and specialized functions in chromatin dynamics (reviewed by Huang et al., 2025). Thus, histone variants define nucleosome properties, while chromatin remodeling complexes reposition or exchange these variant-containing nucleosomes, together regulating chromatin structure and gene expression (Coleman-Derr and Zilberman, 2012; Lang et al., 2012; Stroud et al., 2012; Yelagandula et al., 2014; Lu et al., 2018; Foroozani et al., 2022; Le et al., 2025).

Beyond these mechanisms, RNA modifications (e.g., m^6^A, m^5^C) and higher-order 3D chromatin architecture add further layers of epigenetic regulation with demonstrated roles in plant development and stress responses. RNA modifications occur to modulate the translation of mRNAs, while the spatial organization of the genome within the nucleus governs physical interactions between regulatory elements and genes, respectively. Although these mechanisms are highly relevant to plant trait regulation, they will not be discussed in detail in this review. For more comprehensive coverage, readers are referred to recent reviews on RNA methylation (e.g., Shinde et al., 2023; Shen et al., 2023; Bhat et al., 2025) and 3D genome architecture in plants (Pei et al., 2021; Tourdot and Grob, 2023).

Each type of epigenetic process described plays a role in regulating function and phenotype in plants; indeed, many of these mechanisms are highly conserved among species. While we have a broad scientific foundation in model species such as *Arabidopsis*, it is unclear how easily this knowledge translates to bioenergy crops, many of which are genomically very diverse. As such, in the following section, we suggest deeper investigation is essential uncover how specific traits, functions, and behaviors in bioenergy crops could be enhanced by more intimate knowledge of epigenetics.

## 3 Epigenetic mechanisms regulating important bioenergy crop traits

### 3.1 Growth vigor and biomass yield

Biomass feedstocks include dedicated energy crops (e.g., grasses and woody species), lignocellulosic residues (e.g., maize stover and sorghum stalks), and algae (**Figure 1**). Among these, second-generation lignocellulosic crops such as miscanthus, switchgrass, willow, poplar, and eucalyptus are suitable for sustainable, cost-effective bioenergy due to their high biomass yield (Allwright and Taylor, 2016; Clifton-Brown et al., 2019). Improving yield and adaptability in these crops typically relies on breeding approaches that enhance performance-related traits. One widely used method is hybridization, which can result in heterosis, where hybrids outperform their parents in growth, vigor, and biomass accumulation (Hochholdinger and Hoecker, 2007).

While the genetic basis of heterosis has been extensively studied, epigenetic mechanisms also contribute to phenotypic diversity and hybrid performance without changes to DNA sequence (Tonosaki et al., 2022). In particular, hybridization between epigenetically divergent parents leads to extensive epigenome remodeling, including changes in DNA methylation and small RNA expression, which may influence hybrid vigor even in the absence of classic, heritable epimutations (Groszmann et al., 2013). Epimutations are heritable changes in epigenetic states that give rise to novel epialleles, or specific genes with epigenetic marks that are stably inherited resulting in phenotypic variation. Epimutations induce epialleles that can either occur naturally, driven by environmental or developmental factors, or artificially using chemical agents or targeted tools such as CRISPR/dCas9-based epigenome editing (Tonosaki et al., 2022). These epimutants expand the available pool of functional variation and can affect biomass-related traits, provided they are stable and heritable. For example, paramutation, a well-characterized epigenetic phenomenon in maize, involves one allele inducing a heritable change in its homolog, leading to stable alterations in gene expression across generations (Chandler, 2007; Arteaga-Vazquez and Chandler, 2010). These mechanisms establish a foundation for investigating epigenetic contributions to hybrid performance in bioenergy species, particularly those cultivated as biomass feedstocks.

Hybridization can occur between epigenetically divergent genotypes as well; these genetically similar populations have distinctive epigenetic patterns, either from minor genetic drift or substantial environmental variation. In this case, epigenetic remodeling can occur independently of *de novo* epimutations, contributing to phenotypic variation and heterosis. For example, in *Arabidopsis thaliana*, hybrids of accessions C24 and Landsberg erecta exhibit altered DNA methylation despite nearly identical parental genomes (Meyer et al., 2004). This epigenetic remodeling arises from interactions of divergent parental epigenomes, especially at loci with differing methylation, and involves reduced levels of 24-nucleotide (nt) small interfering RNAs (siRNAs). Consequently, these hybrids display a 250% increase in biomass and enhanced seed yield, demonstrating epigenetic contributions to heterosis (Greaves et al., 2015; Groszmann et al., 2011). Similarly, maize (*Zea mays*) hybrids between inbred lines B73 and Mo17 show downregulation of 24-nt siRNAs at genomic regions differing between parents, notably in developing ears but not in meristematic shoot apices, both of which are essential growth tissues (Barber et al., 2012). These small RNA–mediated epigenetic mechanisms contribute to enhanced overall biomass, including increased maize stover yield, and vigor in maize hybrids (Barber et al., 2012; Ng et al., 2012). Although heterosis for biomass yield has been observed in several bioenergy crops, including switchgrass (Shrestha et al., 2021), miscanthus (Zheng et al., 2022), poplar (McKown and Guy, 2018; Stanton et al., 2019), and willow (Dudits et al., 2022), the epigenetic basis for enhanced yield and vigor in these species remains unexplored and merits further investigation.

A targeted epimutagenesis breeding approach in sorghum exemplifies the potential of implementing these practices to improve bioenergy traits. This strategy involves the manipulation of *MutS HOMOLOG 1* (*MSH1*), a gene involved in organelle genome stability, through plastid-triggered nuclear reprogramming to induce *de novo*, heritable epigenetic changes (Ketumile et al., 2022). When applied to an isogenic sorghum population, combined with early-stage selection, this approach increases biomass yield by 36% under optimal conditions and 64% under marginal field conditions. Transcriptomic profiles of these sorghum epi-lines resemble gene expression changes observed in heterotic Arabidopsis C24 × Landsberg erecta hybrids (Wang et al., 2015), suggesting that perturbation of MSH1 can trigger epigenetic reprogramming with similar downstream transcriptional effects across species. These findings demonstrate that epi-mutation based breeding could serve as a practical complement to conventional hybridization in bioenergy crops, with particular relevance to second-generation lignocellulosic feedstocks where traditional genetic-based improvements may be limited.

### 3.2 Nutrient use efficiency

Efficient nutrient acquisition and utilization are essential for the productivity and sustainability of bioenergy crops, particularly cellulosic crops such as switchgrass, sorghum, and miscanthus, which are often grown on marginal lands with limited inputs (Hattori and Morita, 2010). The environmental advantages of these bioenergy crops can be offset if high fertilizer inputs are required for effective biomass production (Cadoux et al., 2012; Roth et al., 2015). Therefore, improving nutrient use efficiency is required to maximize biomass yield while minimizing input costs and reducing negative environmental impacts. Plants respond to nutrient limitations by reprogramming gene expression, leading to changes in root architecture and physiological processes (López-Bucio et al., 2003). This regulation is not solely transcription factor-dependent: it also involves chromatin-level remodeling, which enables plants to adapt dynamically to nutrient availability (Grimanelli and Roudier, 2013; Zhang et al., 2023). However, our current understanding of epigenetic and chromatin-based mechanisms underlying nutrient adaptation is largely derived from studies in *Arabidopsis thaliana* (reviewed by Séré and Martin, 2020; Zhang et al., 2023).

To extend this understanding to bioenergy plant systems, maize serves as a valuable model due to its high nitrogen requirement. In response to nitrate limitation, the high-affinity nitrate transporter genes *ZmNRT2.1* and *ZmNRT2.2* are upregulated, a process regulated by the chromatin remodeler ZmCHB101, which is a core component of the ATP-dependent SWI/SNF chromatin remodeling complex (Sabermanesh et al., 2017; Meng et al., 2020; Maassen et al., 2025). Under nitrate-deficient conditions, ZmCHB101 binds to nitrate-responsive cis-elements (NREs) within the promoters of *ZmNRT2.1* and *ZmNRT2.2*, maintaining nucleosome occupancy and limiting access by the NIN-like transcription factor, ZmNLP3.1. In the presence of nitrate treatment, ZmCHB101 binding to NREs is significantly reduced, particularly at nucleosomes positioned one or two units upstream or downstream of the NRE site. This reduction in nucleosome density enhances ZmNLP3.1 binding, further increasing the transcription of these transporter genes (Meng et al., 2020). In *ZmCHB101*-RNAi lines, where the chromatin remodeler is knocked down, reduced nucleosome occupancy at the promoters of *ZmNRT2.1* and *ZmNRT2.2* facilitates their transcription, resulting in enhanced root growth and increased biomass compared to wild-type under low nitrogen conditions (Meng et al., 2020), illustrating how a chromatin-based mechanism (via ZmCHB101) represses nitrate uptake genes under low-nitrogen conditions. Further advances on chromatin remodeling in maize and other systems will help epigenetic breeding strategies aimed at optimizing nutrient use efficiency in low nutrient conditions while enhancing biomass production as cellulosic feedstock. In high-input crops like maize, such strategies could reduce fertilizer requirements while maintaining yield. In contrast, low-input perennial bioenergy crops such as switchgrass and miscanthus, though already adapted to nutrient-poor soils, often exhibit reduced biomass productivity under those conditions (Kering et al., 2012; Ding et al., 2021; Hao et al., 2022). Epigenetically enhancing nutrient use efficiency in these species provides a means to optimize—rather than merely tolerate—low-nutrient environments, thereby reducing inputs without compromising yield.

### 3.3 Cell wall composition

The plant cell wall serves as a primary source of lignocellulosic biomass for biofuel production and varies in composition across species, tissue types, and developmental stages (Pauly and Keegstra, 2010). Composed mainly of cellulose, hemicellulose, and lignin, lignocellulosic biomass is deconstructed through pretreatment and enzymatic hydrolysis to release fermentable sugars, which can then be converted into biofuels. However, the proportions and chemical structures of cell wall components differ significantly, influencing conversion efficiency. For instance, poplar (a hardwood) is rich in glucuronoxylans and contains minor mannose-containing polysaccharides, whereas bioenergy grasses (e.g. switchgrass, miscanthus) produce arabinoxylans and lignin-bound p-hydroxycinnamic acids such as p-coumaric and ferulic acid (Loqué et al., 2015). These structural differences influence biomass recalcitrance—the resistance of lignocellulosic biomass to enzymatic deconstruction (Himmel et al., 2007). To process biomass efficiently, current biorefineries rely on chemical pretreatment and enzymatic hydrolysis to release fermentable sugars; however, for economic viability, both biomass yield and processability must be optimized (Somerville et al., 2010). Recent advances in plant biotechnology have shown success in modifying lignin to reduce recalcitrance, minimize fermentation inhibitors, and enhance sugar release without severely compromising growth (Pu et al., 2013; Furtado et al., 2014).

**Figure 2:**
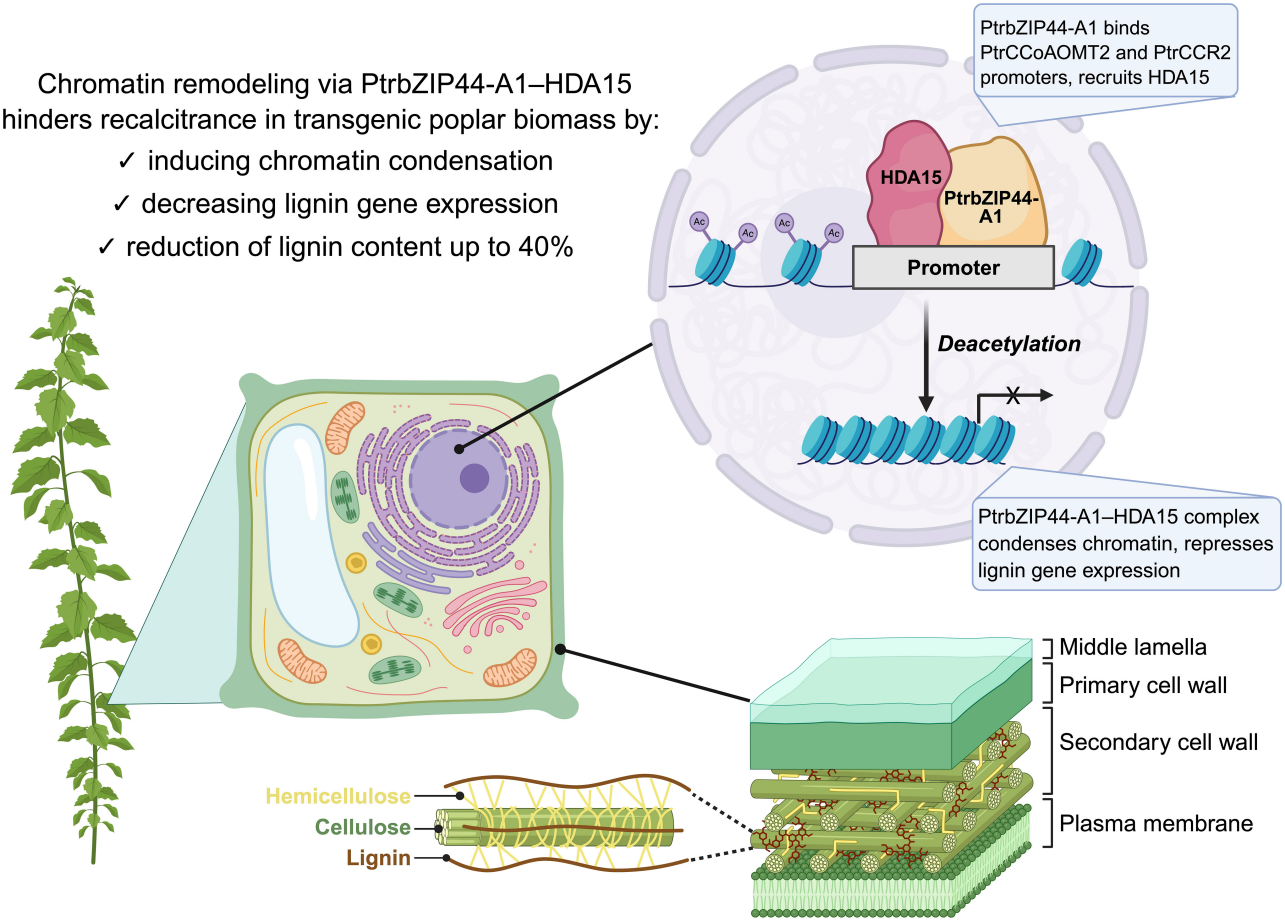
Epigenetic regulation of lignin biosynthesis in *Populus trichocarpa* by PtrbZIP44-A1 and PtrHDA15. PtrbZIP44-A1 binds to the promoters of core lignin biosynthesis genes (*PtrCCoAOMT2* and *PtrCCR2*) and recruits the histone deacetylase PtrHDA15, leading to deacetylation, chromatin condensation, and decreased gene expression. This repression reduces lignin accumulation by up to 40% in transgenic poplar, resulting in lowered biomass recalcitrance and enhanced suitability for bioenergy applications (Ma et al., 2025).

Epigenetic strategies provide a promising means to tailor cell wall composition, enabling targeted lignin modification. A recent discovery in poplar (*Populus trichocarpa*) identified a novel mechanism where the transcription factor PtrbZIP44-A1 binds specifically to two core lignin biosynthesis genes, *PtrCCoAOMT2* and *PtrCCR2*, and recruits the histone deacetylase PtrHDA15 to their promoters (Ma et al., 2025). This interaction induces histone deacetylation, resulting in chromatin condensation and reduced transcription, ultimately lowering lignin accumulation (**Figure 2**). Transgenic lines overexpressing PtrbZIP44-A1 or PtrHDA15 showed reduced lignin (40%), while loss-of-function mutants led to increased lignification, which is comparable to direct gene-silencing approaches (Meyermans et al., 2000; Zhong et al., 2000; Wei et al., 2008; Wang et al., 2012, 2018; Li et al., 2022). Importantly, this pathway demonstrates high specificity, offering a means to directly modulate lignin content without broadly impacting growth. Although constitutive overexpression caused growth retardation, likely due to off-target repression of growth-related genes (Voelker et al., 2010; Mansfield et al., 2012), loss-of-function mutants grew normally, and overexpression enhanced drought tolerance (Li et al., 2019a; Liu et al., 2022; Li et al., 2023; Zhang et al., 2024a). The presence of the ACGTG binding motif (recognized by bZIP transcription factors) in numerous lignin biosynthesis and stress-responsive genes suggests that this regulatory logic may be conserved in other woody bioenergy species. For instance, chromatin-based epigenetic control of lignin genes is also evident in *Eucalyptus grandis*, where lignin biosynthesis genes such as *CAD2* and *CAD3* are marked by activating histone modifications, such as H3K4me3, indicating the importance of chromatin states in regulating cell wall traits (Hussey et al., 2017). These findings suggest that, although epigenetic mechanisms may operate distinctly across taxa, a shared framework of chromatin-level regulation underlies lignin pathway control in diverse bioenergy crops providing a targeted epigenetics-based strategy for developing low-recalcitrance and stress-resilient biomass feedstocks.

### 3.4 Oil biosynthesis

Triacylglycerol (TAG) is the primary form of energy-dense fat stored in plant seeds. This high-energy molecule is essential for seedling development and serves as a valuable biodiesel feedstock, as its fatty acyl chains chemically resemble the aliphatic hydrocarbons found in petroleum-based fuels (Durrett et al., 2008). TAG biosynthesis involves two main stages: *de novo* fatty acid (FA) synthesis in plastids and TAG assembly in the endoplasmic reticulum (Cagliari et al., 2011), both of which are controlled by several gene networks. In oil-producing crops such as camelina (*Camelina sativa*), rapeseed/canola (*Brassica napus*), oil palm (*Elaeis guineensis*), and jatropha (*Jatropha curcas*), both the amount and composition of oil are critical for its biofuel utility, as they determine fuel quality factors like energy content, stability, and combustion efficiency (Murphy, 2012). Phenotypic traits crucial for optimal oil accumulation, such as seed size, fruit development, and oil content, can be epigenetically regulated through the modulation of genes involved in embryo development, lipid metabolism, and the biosynthesis of storage compounds, as illustrated in the following examples from bioenergy crops.

**Figure 3:**
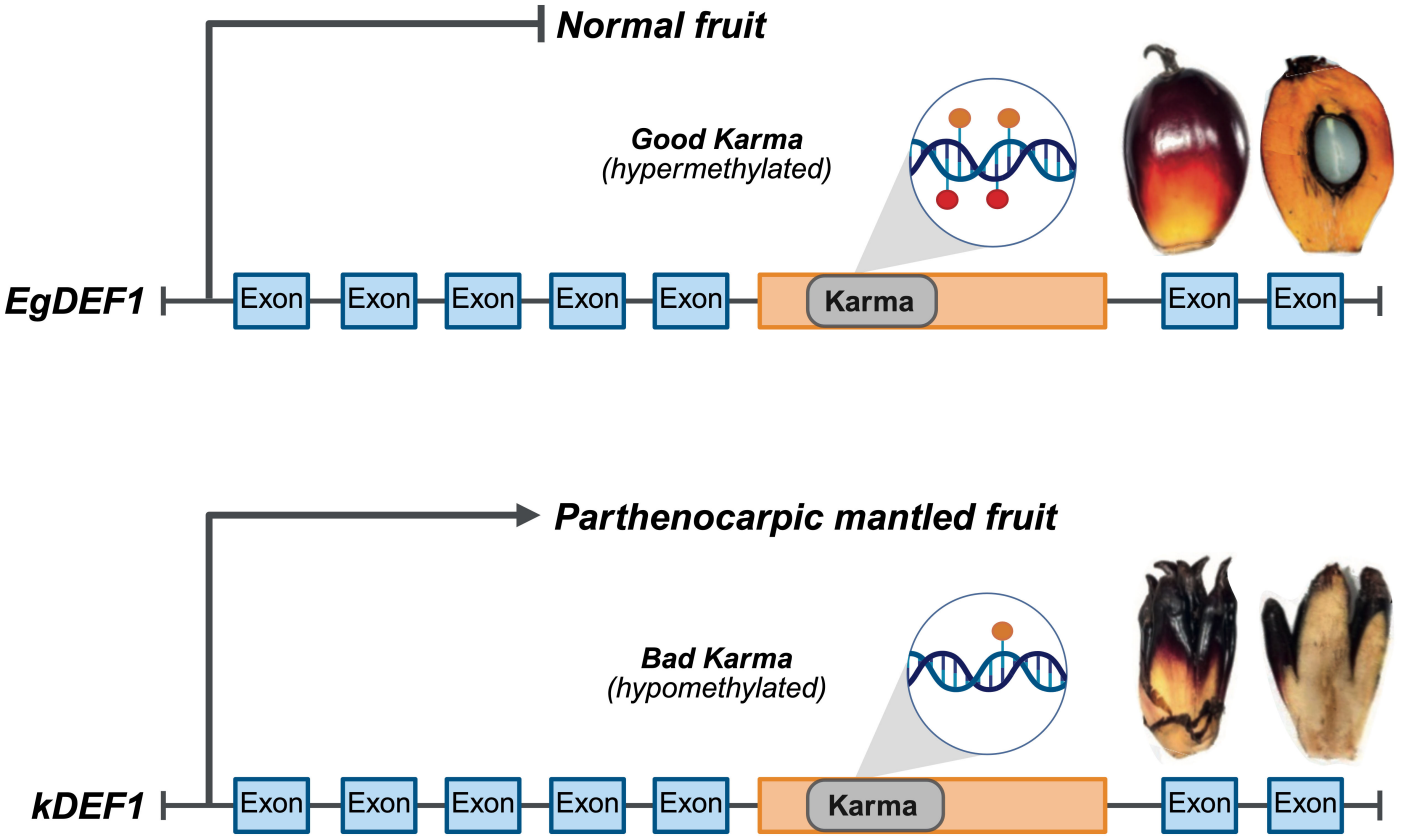
Epigenetic control of the MANTLED locus in oil palm (*Elaeis guineensis*) fruit development. The *EgDEF1* gene contains the Karma LINE retrotransposon (Karma) within intron 5 (orange box). Dense Karma methylation (the “Good Karma” epiallele) maintains normal splicing, suppresses *kDEF1* expression, and results in normal fruit development (top image). In contrast, hypomethylation of Karma (the “Bad Karma” epiallele), common in tissue culture-derived mantled palms, leads to aberrant splicing and expression of the *kDEF1* transcript, resulting in homeotic transformation, parthenocarpy, and reduced yield (bottom image). (Adapted from Sarpan et al., 2020; Fruit images reprinted with permission, license number 6172740192516, from Ong-Abdullah et al., 2015).

African oil palm (*Elaeis guineensis*) is a major source of triacylglycerol used in biofuel production (Alhaji et al., 2024); plant propagation is commonly achieved via tissue culture, enabling rapid multiplication of high-yielding, genetically uniform plants. However, this method often results in a high incidence of an abnormal fruit phenotype known as ‘mantled’ (Mgbeze & Iserhienrhien, 2014). In mantled palms, reproductive organs such as staminodes and stamens undergo homeotic transformation, developing into pseudocarpels—structures resembling carpels but lack full reproductive functionality—which leads to sterile, parthenocarpic flowers with abortive fruit and significantly reduced oil yields (Adam et al., 2007; Mgbeze & Iserhienrhien, 2014). An epigenome-wide association study (EWAS) linked the mantled phenotype to DNA hypomethylation of a LINE retrotransposon (Karma) located in an intron of the homeotic gene *DEFICIENS* (*EgDEF1*), which encodes a B-type MADS-box transcription factor (**Figure 3**). This loss of methylation at the Karma splice site (the “Bad Karma” allele) leads to aberrant splicing, premature transcription termination, and subsequent expression of the isoform, *kDEF1*, in the flowers of mantled clones (**Figure 3**). Conversely, dense methylation (the “Good Karma” epiallele) maintains normal gene expression and normal fruit development, while Bad Karma induces homeotic transformations, parthenocarpy, and dramatic yield loss (Ong-Abdullah et al., 2015). This discovery enabled screening of oil palm clones for aberrant epigenetic modifications prior to planting, a strategy that has dramatically reduced the occurrence of the mantled phenotype and enhanced yield and productivity. This discovery and its successful implementation toward improving palm oil productivity, and therefore stabilizing TAG production, exemplifies how epigenetic knowledge can be directly harnessed to improve crop performance. It underscores the importance of deepening our understanding of epigenetic regulation of crop traits, particularly for the bioenergy sector, in which maximizing yield and minimizing instability are critical for economic sustainability. Yet, despite this and other promising examples, the widespread adoption of epigenetic strategies in bioenergy crops remains limited.

Building on this, the bioenergy species jatropha (*Jatropha curcas*) offers another compelling example for exploring epigenetic regulation of oil production and accumulation. Studies demonstrate functional conservation of several enzymes involved in fatty acid synthesis, including the enzyme diacylglycerol acyltransferase 1 (*JcDGAT1*) which enhances flux toward TAG synthesis, among model and non-model species like *Arabidopsis* and *Jatropha.* Overexpression of JcDGAT1 in *Arabidopsis*, either constitutively or under seed-specific promoters, increases seed oil content up to 41% without adverse phenotypic effects (Misra et al., 2013). Harnessing this conserved genetic basis for seed oil accumulation in bioenergy crops, particularly the epigenetic processes that may govern its utilization, remains comparatively understudied. Indeed, *Arabidopsis thaliana* employs several chromatin-based mechanisms to modulate seed oil biosynthesis: the histone methyltransferase CURLY LEAF (CLF), part of the Polycomb Repressive Complex 2 (PRC2), deposits the repressive mark H3K27me3 on fatty acid metabolism genes (Liu et al., 2016). Mutants of *clf* exhibit increased seed size, elevated oil content, and altered fatty acid profiles, suggesting that CLF represses oil biosynthesis during embryogenesis. Similarly, the chromatin remodeler PICKLE (PKL) suppresses transcription factors, such as LEC1, LEC2, and FUS3, that regulate seed maturation and lipid accumulation. In *pkl* mutants, ectopic activation of these transcription factors leads to lipid accumulation in non-seed tissues (Ogas et al., 1999; Zhang et al., 2012). Although direct evidence for epigenetic regulation of triacylglycerol biosynthesis in *Jatropha curcas* is limited, the conservation of biosynthetic pathways supports the hypothesis that analogous epigenetic mechanisms may operate in jatropha and other oilseed bioenergy crops (Ma et al., 2017). Investigating these regulatory layers could reveal novel targets for expanding the epigenetic toolkit for improving feedstocks, particularly as the field moves beyond conventional breeding and transgenic approaches.

Other purpose-grown feedstocks, including green microalgae (Chlorophyta), naturally accumulate TAGs and often exhibit higher lipid content than many traditional oilseed crops; as such, they are valuable candidates for biodiesel production (Wang et al., 2024). However, high biomass does not necessarily correlate with high lipid productivity, and increased lipid content can reduce overall biomass yield. Therefore, optimizing lipid productivity requires balancing both lipid content and biomass. Under stress conditions, many microalgae redirect lipid biosynthesis toward neutral lipids, primarily TAGs (Venkata Mohan et al., 2011; Prathima Devi et al., 2013). Epigenetic modifications, particularly DNA methylation, are pivotal in regulating microalgal stress responses and lipid accumulation (Ngan et al., 2015; Kronholm et al., 2017; Steadman, 2023; Steadman et al., 2020). In the biofuel-relevant species *Picochlorum soloecismus*, nitrogen starvation induces global DNA hypomethylation, which leads to changes in the expression of genes involved in metabolic pathways. This epigenetic reprogramming shifts cellular metabolism towards enhanced lipid biosynthesis, a form of carbon storage that helps the organism survive nutrient stress and nitrogen depletion. Supporting this, chemical inhibition of DNA methylation using 5-aza-2′-deoxycytidine enhances lipid accumulation by approximately 66%, demonstrating that reduced DNA methylation directly influences gene regulation pathways controlling carbon partitioning and lipid storage in response to nitrogen limitation (Steadman et al., 2020). Manipulating the DNA methylome in microalgae may thus provide a strategy to optimize lipid accumulation for biofuel production. More broadly, understanding and engineering the epigenetic regulation of lipid metabolism in both microalgae and oilseed crops could accelerate the development of high-yielding, sustainable biofuel feedstocks.

### 3.5 Abiotic stress tolerance

Epigenetic regulation of abiotic stress responses, including extreme temperatures, drought, salinity, heavy metal toxicity, and nutrient deficiency, involves a layered and interconnected network of mechanisms (reviewed by Sudan et al., 2018; Chang et al., 2020; Abdulraheem et al., 2024; Jin et al., 2024; Shilpa et al., 2024; Ma et al., 2025). Central to these mechanisms are DNA methylation and chromatin modifications, which are interdependent and regulated in part by RNA interference (RNAi)-based pathways (Saze et al., 2012). Simplistically, histone modifications influence chromatin structure and transcriptional activity, while DNA methylation often provides more stable and long-term transcriptional control. Additionally, the RdDM pathway facilitates *de novo* methylation via siRNAs (Saze et al., 2012) for enhanced adaptation. Together, these mechanisms coordinate the expression of stress response genes, enabling plants to adapt to environmental challenges. Given that abiotic stresses threaten agricultural productivity, understanding their epigenetic regulation is critical across all crop species, including bioenergy crops.

Drought stress triggers dynamic histone acetylation remodeling at drought-responsive loci, mediated by the antagonistic activities of HATs and HDACs. These enzymes modulate chromatin accessibility within abscisic acid (ABA) and jasmonic acid (JA) signaling pathways by targeting specific promoter and enhancer regions, often in coordination with transcription factors and co-regulators (Li et al., 2021). Convergent yet species-specific epigenetic regulation strategies in response to drought stress are evident from multi-omics studies examining histone modification dynamics, as demonstrated by the following examples in bioenergy crops.

**Figure 4:**
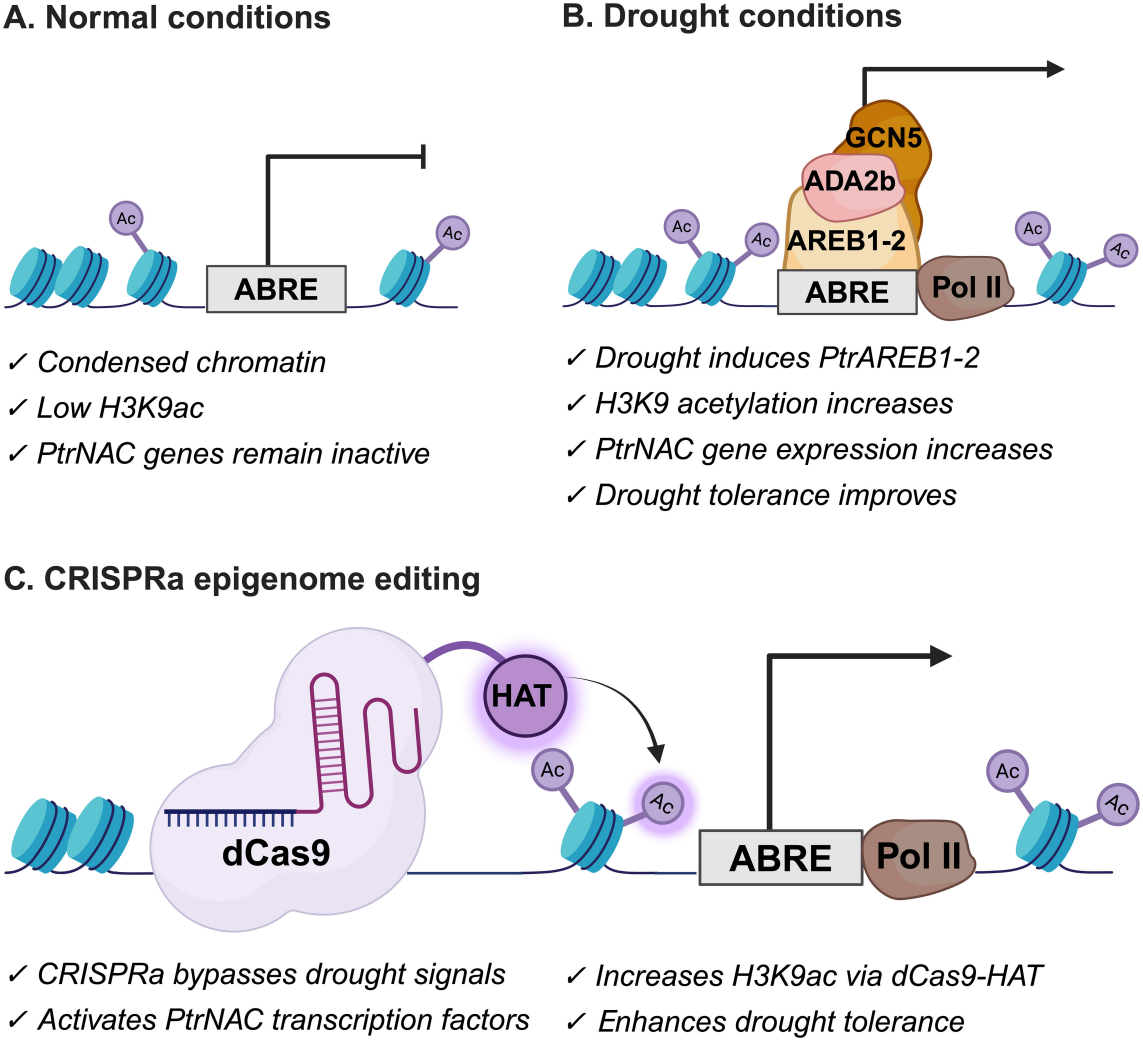
Models illustrating regulation of drought tolerance in *Populus trichocarpa* and proposed CRISPRa-mediated epigenome editing strategy. **(A)** Normal conditions: Chromatin at *PtrNAC* gene loci is relatively condensed with low H3K9 acetylation, resulting in minimal *PtrNAC* expression. **(B)** Drought conditions: Drought stress activates ABA signaling, leading to activation of the ABRE-binding transcription factor PtrAREB1–2. PtrAREB1–2 binds ABA-Responsive Element (ABRE) motifs in the promoters of drought-responsive *PtrNAC* genes and recruits the histone acetyltransferase complex ADA2b–GCN5. This recruitment increases H3K9 acetylation, promotes RNA polymerase II enrichment, and activates *PtrNAC* gene expression, thereby enhancing drought tolerance. The drought-responsive *PtrNAC* genes include *PtrNAC006, PtrNAC007*, and *PtrNAC120* (adapted from Li et al., 2019a; Castroverde, 2019). **(C)** Proposed CRISPRa epigenome editing strategy in poplar based on *Arabidopsis*: A nuclease-dead Cas9 (dCas9) fused with a histone acetyltransferase (HAT) and guided by a single guide RNA (sgRNA) bind to *PtrNAC* gene loci. Upon dCas9-HAT targeting, the HAT catalyzes histone acetylation, leading to local chromatin relaxation. This chromatin opening enhances transcriptional machinery at the *PtrNAC* target locus, thereby activating gene expression (Adapted from Roca Paixão et al., 2019 and de Melo et al., 2020).

In *Populus trichocarpa*, integrative chromatin immunoprecipitation sequencing (ChIP-seq), a technique used to map specific protein–DNA interactions, combined with RNA sequencing (RNA-seq) to assess gene expression under drought stress, identified *PtrNAC006, PtrNAC007*, and *PtrNAC120* as key drought-responsive genes based on increased expression compared to normal conditions (**Figure 4A**). These drought responsive genes were marked by increased, a histone modification associated with gene activation, specifically at abscisic acid (ABA)-responsive element (ABRE)-containing promoters (Li et al., 2019a). ABA is a phytohormone that mediates adaptive responses to drought by regulating stomatal closure, osmotic balance, and stress-responsive gene expression, making it an important target for drought resilience strategies (Muhammad Aslam et al., 2022). During drought conditions, the ABRE motif in the promoter of drought-responsive genes is bound by the ABA-inducible transcription factor PtrAREB1-2, which recruits the ADA2b–GCN5 histone acetyltransferase complex, enhancing H3K9ac deposition and RNA polymerase II recruitment (**Figure 4B**). Promoter motif analysis across the genome further identified 76 transcription factors with ABRE motifs—including 11 NAC (NAM, ATAF1/2, and CUC2) homologs, a family of plant-specific transcription factors involved in stress responses and developmental regulation—supporting a broader regulatory network. Overexpression of *PtrNAC006*, *PtrNAC007*, and *PtrNAC120* led to significantly improved drought survival (~76%, ~56%, and ~39%, respectively) compared to wild-type plants (~13%) (Li et al., 2019a). A similar approach in *Sorghum bicolor* revealed differential enrichment of five permissive histone marks including those involved with gene activation (H3K9ac and H3K27ac) and transcription (H3K4me3, H3K4me2, and histone variant H2A.Z) at drought-responsive genes, particularly clade A *PP2C* genes, which are negative regulators of ABA signaling. Many loci exhibited combinatorial enrichment of activating marks, suggesting a multilayered chromatin signature facilitating transcriptional activation. Promoter motif analysis identified enrichment of Ethylene Response Factor (ERF) binding motifs at these genes, implicating ERF transcription factors as mediators of epigenetic remodeling in sorghum drought responses (Hu et al., 2025). ERFs are a large family of plant-specific transcription factors that regulate gene expression in response to various abiotic stresses (Wu et al., 2022). Their recruitment to chromatin marked by activating histone modifications suggests ERFs may help establish or maintain permissive chromatin states under drought stress, integrating epigenetic and transcriptional control for optimized stress resilience.

While chromatin landscapes and transcription factor networks vary by species, a theme across these studies is the integration of ABA signaling with dynamic histone modifications to regulate drought-responsive gene expression, thus providing an avenue for improving drought resilience through epigenome-informed breeding and engineering. This strategy has been applied in *Arabidopsis thaliana*, where activation of the *ABA-responsive element binding protein 1* (*AREB1*) using a CRISPRa system fused to a histone acetyltransferase (dCas9-HAT1) led to enhanced drought tolerance in *Arabidopsis*, characterized by increased expression of downstream stress-responsive genes like *Response to Desiccation 29A* (*RD29A*), higher chlorophyll content, faster stomatal response, and improved survival under drought stress (Roca Paixão et al., 2019). Survival assays revealed that 85% of transgenic plants recovered after short-duration stress, compared to 50% of controls, while 100% survived after mild severe drought stress, versus complete mortality in controls. In *Populus trichocarpa*, a similar CRISPRa strategy could be employed to directly activate *PtrNAC* genes by recruiting histone acetyltransferase activity to their promoters, thereby enhancing drought tolerance independently of upstream ABA signaling (**Figure 4C**). This approach would effectively demonstrate how targeted chromatin remodeling can enhance abiotic stress tolerance and provide translational applications for other bioenergy crops.

### 3.6 Biotic stress tolerance

In addition to abiotic stresses, plants encounter numerous pathogens that require rapid comprehensive responses given their sessile nature. Across diverse plant–pathogen systems, salicylic acid (SA), jasmonic acid (JA), auxin, and other hormones are fundamental to plant defense, and epigenetic processes consistently regulate these hormonal pathways (Tao et al., 2023; Xie and Duan, 2023). This intersection of hormone signaling and epigenetic control emerges as a unifying mechanism for dynamic, precise regulation of plant defense. Studies in bioenergy crops demonstrate that these epigenetic pathways are functionally relevant—as detailed below—and have the potential to enhance disease resistance, thereby minimizing chemical inputs, and improving yield.

Gibberella stalk rot (GSR), caused by *Fusarium graminearum*, is a major fungal disease in maize, causing significant yield losses; recent discoveries have identified the gene *ZmCCT* as a significant regulator of resistance through an epigenetically controlled defense mechanism (Wang et al., 2017). *ZmCCT* is located at the major resistance quantitative trait locus (QTL) known as *qRfg1*. Its activity is influenced by the presence of a CACTA-like transposable element (TE1) in its promoter, which disrupts a poised chromatin state, characterized by both active (H3K4me3) and repressive (H3K27me3/H3K9me3) histone marks. This disruption reduces H3K4me3 levels and increases DNA methylation, making the gene unresponsive to pathogen attack and leading to susceptibility (Wang et al., 2017). In contrast, the non-TE1 allele (Y331-ΔTE1) undergoes rapid and transient transcriptional activation within three hours post-inoculation, triggering early immune responses while maintaining low basal expression under normal conditions. A multi-omics study further revealed that *ZmCCT* likely influences defense responses through hormone signaling, particularly the SA and auxin pathways, which contribute directly to pattern-triggered immunity (PTI) in resistant lines (Tang et al., 2022). In Y331-ΔTE1 plants, early activation of genes in auxin and SA signaling was observed alongside changes in metabolic pathways including phenylpropanoid and phenylalanine metabolism, supporting the idea that *ZmCCT* primes hormone-responsive defense networks during the early biotrophic stage of infection (Hao et al., 2018; Bari and Jones, 2009). Experimental evidence confirms elevated auxin signaling in resistant roots, suggesting that *ZmCCT* contributes to GSR resistance by regulating hormonal responses. *ZmCCT* exhibits tissue-specific expression, mediating photoperiod sensitivity in leaves and defense activation in roots, making it an ideal candidate for epigenetic editing to decouple developmental and immune functions (Wang et al., 2017).

*Botrytis cinerea*, a necrotrophic fungus, that causes grey mold in *Brassica napus* (canola) exploits hormonal crosstalk in plants by actively inducing the SA pathway. Typically, the SA pathway mediates resistance against biotrophic pathogens by activating SA-specific defense genes that trigger hypersensitive responses, effectively restricting pathogen spread. In contrast, the JA pathway is primarily activated against necrotrophic pathogens, promoting JA-responsive genes that suppress the hypersensitive response to prevent excessive cell death (Klemme et al., 2019). However, by inducing the SA pathway, *B. cinerea* promotes hypersensitive cell death, which facilitates its colonization while simultaneously suppressing the JA pathway (Klemme et al., 2019). To investigate how epigenetic regulation influences this hormonal balance, SA tolerant epilines (lines with heritable epigenetic modifications rather than genetic sequence changes) were generated from an isogenic canola background that exhibited reduced sensitivity to SA (Klemme et al., 2019). These SA-tolerant epilines showed altered transcriptional profiles of canonical and novel SA-responsive genes upon SA treatment, indicating a reprogrammed defense network. Notably, the SA-tolerant line with the lowest SA-induced gene expression demonstrated enhanced tolerance to *Botrytis cinerea*, which correlated with reduced levels of the activating histone modification H3K4me3 at genes that initiate the SA pathway. Collectively, these studies demonstrate that hormone-mediated defense pathways are epigenetically regulated in diverse bioenergy crops and could be targeted through epigenomic editing to enhance pathogen resistance and, in turn, improve disease resilience and crop productivity.

## 4 Emerging epigenetic technologies for bioenergy crops

**Figure 5:**
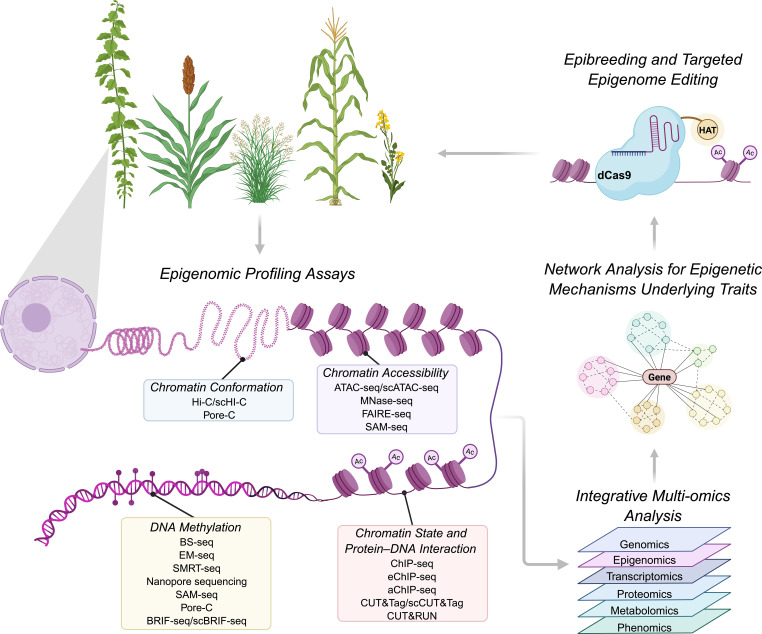
Integration of epigenomic technologies for bioenergy crop improvement. An illustrated pipeline from bioenergy crops through relevant epigenomic profiling techniques and multi-omics analyses to uncover regulatory mechanisms and guide trait improvement. Chromatin conformation is analyzed using high-throughput chromosome conformation capture (Hi-C), single-cell Hi-C (scHi-C), and Pore-C. Chromatin accessibility is profiled with assay for transposase-accessible chromatin using sequencing (ATAC-seq), single-cell ATAC-seq (scATAC-seq), micrococcal nuclease sequencing (MNase-seq), formaldehyde-assisted isolation of regulatory elements sequencing (FAIRE-seq), and Simultaneous Accessibility and Methylation Sequencing (SAM-seq). Chromatin state and protein-DNA interactions are assessed using chromatin immunoprecipitation sequencing (ChIP-seq),Enhanced ChIP (eChIP), advanced ChIP (aChIP), cleavage under targets and tagmentation (CUT&Tag), single-cell CUT&Tag (scCUT&Tag) and cleavage under targets and release using nuclease (CUT&RUN). DNA methylation is examined with bisulfite sequencing (BS-seq), enzymatic methyl-seq (EM-seq), Pacific Biosciences (PacBio) single-molecule real-time (SMRT) sequencing, Oxford Nanopore Technologies (ONT) sequencing, SAM-seq, Pore-C, bisulfite-converted randomly integrated fragments sequencing (BRIF-seq) and single-cell BRIF-seq (scBRIF-seq). Data from these epigenomic analyses are integrated with multi-omics layers including phenomics, metabolomics, proteomics, transcriptomics, and genomics to identify epigenetic regulatory mechanisms underlying bioenergy-relevant traits. Insights from these analyses guide targeted epigenome editing and epi-breeding strategies using CRISPR–dCas9-based systems for stable modulation of gene expression and creation of heritable epialleles. Edited plants can be cycled back into breeding programs to accelerate the development of improved bioenergy crop cultivars or used for functional validation of candidate regulatory elements to inform breeding strategies.

Recent advancements in technologies like high-throughput epigenomic profiling, CRISPR/Cas-based epigenome editing and single-cell epigenomics have significantly expanded our understanding of epigenetic mechanisms and their impact on cellular function. These technologies also facilitate the study of 3D genomics, which involves examining chromatin interactions and the three-dimensional spatial organization of the genome within the nucleus, as well as how this organization influences gene transcription, DNA replication, repair, and other biological processes in conjunction with linear genome sequence information (Lanctôt et al., 2007; Dekker et al., 2017; Pei et al., 2021). Although some of these technologies are still in their infancy in plant research, they hold significant promise for enhancing our ability to uncover epigenetically regulated traits essential to bioenergy crops and utilize identified regulatory mechanisms to guide functional validation and breeding strategies (**Figure 5**).

### 4.1 Epigenomic profiling

DNA methylation analysis has traditionally relied on bisulfite sequencing (BS-seq), which offers single-base resolution but causes severe DNA damage, sequence biases, and requires extensive PCR amplification, limiting its use in long-read sequencing applications (Leduque et al., 2024). To overcome these drawbacks, enzymatic methyl-seq (EM-seq) has been developed as a gentler alternative that enzymatically converts unmethylated cytosines to uracils without damaging the DNA backbone. EM-seq is optimized for Illumina short-read platforms and provides higher mapping rates, lower duplication, and improved detection of methylation in challenging sequence contexts such as CHG and CHH (Feng et al., 2020).

Building on these biochemical advances, long-read sequencing technologies have emerged as powerful tools for comprehensive methylation mapping across large, repeat-rich plant genomes (Agius et al., 2023). Pacific Biosciences (PacBio) single-molecule real-time (SMRT) sequencing detects DNA methylation through alterations in polymerase kinetics during sequencing, delivering exceptionally high read accuracy (>99.9%) but requires high-quality input DNA and deep coverage (Chen et al., 2022; Qi et al., 2022; Ni et al., 2023). Oxford Nanopore Technologies (ONT), in contrast, enables direct detection of base modifications by sensing disruptions in electrical current as native DNA passes through nanopores that are characteristic of specific modifications (Liu et al., 2019; Ni et al., 2019, 2021; Colicchio et al., 2023; Steadman et al., 2025). ONT generates ultra-long reads ideal for haplotype-resolved methylation profiling and complex genomic regions, which is particularly advantageous in polyploid or heterozygous plant genomes (Liu et al., 2019; Akbari et al., 2021; Ding and Chen, 2018). Despite lower raw read accuracy (generally below 90%), and its reliance on sophisticated computational models trained on known methylation patterns (Logsdon et al., 2020; Chen et al., 2022; Ni et al., 2023; Steadman et al., 2025), the portability and minimal infrastructure demands of ONT make it well-suited for high-throughput and field-based epigenomic studies (Zhang et al., 2018).

In addition to methylation profiling, chromatin accessibility and chromatin state mapping are essential for understanding regulatory landscapes across tissues, developmental stages, and environments (Candela-Ferre et al., 2024). Chromatin accessibility profiling methods include Formaldehyde-Assisted Isolation of Regulatory Elements sequencing (FAIRE-seq), which isolates nucleosome-free DNA via phenol–chloroform extraction, providing a simple, cost-effective, and non-enzymatic approach for mapping open chromatin regions (Baum et al., 2020). Micrococcal Nuclease sequencing (MNase-seq) uses controlled enzymatic digestion to precisely define nucleosome positions across the genome by selectively cleaving linker DNA between nucleosomes. The resulting mononucleosomal DNA fragments are purified and sequenced to infer nucleosome occupancy and positioning with base-pair resolution (Zhang and Jiang, 2018). The Assay for Transposase-Accessible Chromatin using sequencing (ATAC-seq) employs a hyperactive Tn5 transposase to simultaneously fragment and tag accessible DNA, enabling rapid library preparation and low-input applications (Buenrostro et al., 2013). Given its simplicity, sensitivity, and compatibility with single-cell platforms, ATAC-seq has become the most widely used method for profiling chromatin accessibility in plants (Bajic et al., 2018).

While chromatin immunoprecipitation followed by sequencing (ChIP-seq) has been widely used to map DNA–protein binding, it suffers from high background and artifacts due to cross-linking and chromatin solubilization (Kharchenko et al., 2008). In plants, these challenges are compounded by rigid cell walls and complex tissue matrices, leading to low chromatin recovery. Enhanced ChIP (eChIP) and advanced ChIP (aChIP) were developed to overcome these limitations, enabling efficient chromatin profiling in both vegetative tissues and complex economically-relevant organs such as seeds and fruits, thereby extending ChIP-based analyses to a broader range of plant systems (Zhao et al., 2020; Zhang et al., 2024b). More recently, enzyme-tethering strategies such as Cleavage Under Targets and Release Using Nuclease (CUT&RUN) and Cleavage Under Targets and Tagmentation (CUT&Tag) have emerged as high-resolution alternatives to ChIP-seq. These methods profile DNA–protein interactions *in situ* without cross-linking, greatly reducing background noise and input requirements (Skene et al., 2018; Kaya-Okur et al., 2019). CUT&RUN uses a micrococcal nuclease to release targeted DNA fragments, whereas CUT&Tag employs a Tn5 transposase to simultaneously fragment and tag DNA, enabling rapid library preparation and improved signal specificity, with recent adaptations demonstrating effectiveness in plant systems such as *Arabidopsis* and cotton (Zheng et al., 2019; Tao et al., 2020).

Recent innovations combine these layers of epigenomic information within single molecules. For example, ONT-based Simultaneous Accessibility and Methylation Sequencing (SAM-seq) enables concurrent detection of DNA methylation and chromatin accessibility, including nucleosome positioning (Leduque et al., 2024). Additionally, Pore-C integrates long-read sequencing with chromatin conformation capture to resolve three-dimensional genome architecture, providing insights into higher-order regulation (Li et al., 2022).

These technological and biochemical innovations hold promise for bioenergy crop research, where complex, large, and often polyploid genomes present significant challenges. By enabling precise mapping of methylation patterns and chromatin states across native, intact DNA molecules, these methods facilitate more accurate, scalable, and multidimensional views of genome regulation. Applications to identify epigenetic regulatory elements through epigenomic profiling linked to bioenergy-relevant traits include:

- Differential methylation or chromatin accessibility between phenotypes or tissues to uncover trait-associated regulatory elements.
- Correlation of methylation states with gene expression to reveal epigenetic control of specific biological pathways.
- Population-scale methylation profiling to define stable epialleles underlying phenotypic variation.
- Chromatin state dynamics during development and environmental responses to uncover regulatory mechanisms of trait plasticity.
- Transposable element activity and silencing to understand their impact on genome stability and regulation.
- Epigenetic reprogramming during hybridization or polyploidy events to elucidate regulatory changes affecting inheritance.
- Robust, trait-linked methylation markers (e.g., DMRs) for use in breeding and selection programs.

### 4.2 Single-cell epigenomics

Single-cell ‘omics technologies enable detailed characterization of plant tissues by capturing diverse molecular features such as gene expression, chromatin accessibility, DNA methylation, and chromatin conformation at the single-cell resolution. These approaches support the analysis of cellular heterogeneity and regulatory processes across complex tissue types (Yu et al., 2023). Recent studies have utilized single-cell RNA-sequencing (scRNA-seq) approaches in bioenergy crops, including poplar and sorghum, providing high-resolution insights into cell-type-specific gene expression and regulatory mechanisms underlying biomass production and stress responses, respectively (Chen et al., 2021; Fu et al., 2024; Schmidt et al., 2025). While single-cell epigenomic profiling technologies for plants remains in the early stages of development, ongoing advancements are expected to expand high-resolution epigenetic profiling to bioenergy crops. Currently, these technologies have been applied to a handful of plant species: *Arabidopsis thaliana* (*Arabidopsis*) (Farmer et al., 2021; Dorrity et al., 2021), *Oryza sativa* (rice) (Zhou et al., 2019; Ouyang et al., 2021; Feng, 2022), and *Zea mays* (maize) (Li et al., 2019b; Marand et al., 2021).

Among single-cell epigenomic technologies, single-cell bisulfite sequencing (scBS-seq) was initially developed to profile DNA methylation heterogeneity at single-cell resolution in mammalian systems (Smallwood et al., 2014). However, scBS-seq often under-represents highly methylated regions, which tend to form long fragments that are difficult to sequence efficiently. To overcome this limitation, single-cell bisulfite-converted randomly integrated fragments sequencing (scBRIF-seq) was developed, combining bisulfite conversion, random priming, fragment ligation, and multiple displacement amplification to achieve higher read mapping rates and more uniform genome coverage (Li et al., 2019b). When applied to maize microspores, which have a highly methylated and repetitive genome, scBRIF-seq provided comprehensive coverage across CG, CHG, and CHH contexts and revealed both intra-tetrad consistency and inter-tetrad variation in methylation states, indicating asynchronous DNA methylation reprogramming during male gametogenesis (Li et al., 2019b). Importantly, scBRIF-seq detected heterogeneity largely independent of genetic background and preferentially in genic regions rather than transposable elements, demonstrating its utility for studying epigenetic reprogramming, methylation dynamics, and cell-to-cell variation in plant genomes (Li et al., 2019b).

Single-cell ATAC-seq (scATAC-seq) enables chromatin accessibility profiling at single-cell resolution, uncovering cell-type-specific regulatory landscapes and dynamic chromatin states during development. It has been successfully applied to maize organs Marand et al., 2021; Zhang et al., 2024c) and *Arabidopsis* roots (Dorrity et al., 2021). A recent high-throughput variant, scifi-ATAC-seq, pre-indexes nuclei with dual-barcoded Tn5 prior to droplet encapsulation, increasing throughput more than twentyfold without compromising data quality (Zhang et al., 2024c). This scalability facilitates the discovery of rare cell types and genotype-specific regulatory elements, advancing our ability to connect chromatin accessibility with developmental and adaptive traits in crops.

Complementing chromatin accessibility profiling, single-nucleus chromatin immunocleavage with sequencing (snCUT&Tag; referred to as scCUT&Tag for simplicity in **Figure 5**) has been developed for plants to profile histone modifications to dissect epigenetic heterogeneity at the single-cell level. Unlike traditional methods limited by plant cell walls, snCUT&Tag combines antibody-guided chromatin cleavage with droplet-based single-nucleus barcoding enabling robust detection of histone marks (Ouyang et al., 2021; Ouyang et al., 2022). When applied to rice seedlings, this approach profiled H3K4me3 enrichment and partitioned nuclei into 17 distinct cell clusters, each defined by characteristic chromatin signatures at marker genes. Integration with enhancer–promoter interaction models and Chromatin Interaction Analysis by Paired-End Tag sequencing (ChIA-PET), which maps protein-mediated chromatin loops genome-wide, revealed strong concordance with active regulatory contacts and demonstrated clear cell-type-specific chromatin interactions. Over half of the predicted enhancers overlapped accessible chromatin regions identified by ATAC-seq (Ouyang et al., 2022). These results highlight snCUT&Tag’s ability to annotate regulatory elements, reconstruct 3D genome structures, and elucidate the chromatin basis of cell-type-specific regulation in plants.

High-throughput chromosome conformation capture (Hi-C) is a technique developed to capture long-range DNA-DNA interactions to study the 3D structure of the genome. Single-cell Hi-C (scHi-C) facilitates the investigation of 3D genome architecture, revealing how chromatin organization influences gene regulation at cellular resolution. Single-cell Hi-C of rice gametes and zygotes revealed distinct chromosomal compartment folding and spatial arrangements compared to mammals, characterized by shorter-range chromatin contacts and discrete territories associated with specific histone marks and gene activity (Zhou et al., 2019). Furthermore, this technique demonstrated that A/B chromatin compartmentalization is present in rice gametes and likely inherited by the zygote, contributing to rapid zygotic genome activation after fertilization (Zhou et al., 2019). Dynamic reorganization of sperm and egg 3D genomes post-fertilization demonstrates scHi-C’s utility in uncovering chromatin-based regulatory mechanisms underlying epigenetic reprogramming and developmental transitions in plants.

Single-cell epigenomic profiling maps cell-type-specific regulators and chromatin dynamics in cellular specialization and stress responses; there are several ways in which these methods could be applied to developing a deeper understanding of epigenetic regulation of bioenergy crops for enhanced performance, as follows:

- Dissect cell-type-specific chromatin and methylation landscapes in complex tissues to reveal regulatory elements driving trait expression.
- Integrate single-cell transcriptomes with epigenomic profiles (e.g., scATAC-seq, scCUT&Tag) to uncover gene regulatory networks and identify novel cell-type-specific regulators.
- Track cellular developmental trajectories and chromatin state transitions to understand how epigenetic dynamics control cell differentiation and stress adaptation.
- Analyze rare or transient cell populations with high-resolution omics to pinpoint specialized regulatory mechanisms relevant to bioenergy crop traits.
- Capture epigenetic heterogeneity among cells to explain phenotypic plasticity and variability within bioenergy crop populations.
- Map 3D chromatin architecture and enhancer-promoter interactions at single-cell resolution, revealing higher-order regulatory mechanisms shaping traits.
- Leverage spatial transcriptomics and emerging spatial omics to correlate epigenomic states with tissue architecture and environmental responses *in situ*.
- Develop single-cell-resolved epigenetic markers to facilitate precision breeding targeting cellular-level regulatory variation.

### 4.3 CRISPR-based epigenome editing

CRISPR-based epigenome editing offers a precise and adaptable approach to improving bioenergy crop traits when gene targets are known (Zhang and Zhu, 2025). This technology employs a catalytically inactive Cas9 (dCas9) protein guided by customizable RNAs to specific genomic sites (Qi et al., 2013). Unlike traditional systems like zinc fingers or TALENs, CRISPR–dCas9 is easily retargeted by altering guide RNA sequences (Gao et al., 2014; Thakore et al., 2016; Nakamura et al., 2021).

When fused to chromatin-modifying effectors, dCas9 can activate or repress genes by altering DNA methylation or histone modifications (Shin et al., 2022). Repression is typically achieved using domains like KRAB, which promote heterochromatin formation (Thakore et al., 2015), while activation can be driven by domains such as VP64, p300, or VPR (Chavez et al., 2015; Hilton et al., 2015; Lowder et al., 2015). Furthermore, dCas9 fusions with enzymes like TET1, MQ1, or DRM proteins enable targeted editing of DNA methylation marks, resulting in stable gene activation or silencing (Gallego-Bartolomé et al., 2018; Ghoshal et al., 2021). Amplification systems such as SunTag and MS2 further enhance editing efficiency by recruiting multiple effector proteins and transcription factors (Tanenbaum et al., 2014; Lee et al., 2019).

Although many of these systems were initially developed in animal models, their application in plants is now rapidly expanding (Qi et al., 2023; Baldini et al., 2025). CRISPRi (CRISPR interference) and CRISPRa (CRISPR activation) systems are tunable and can target specific alleles in polyploid genomes either independently or simultaneously, enabling precise dosage control, which is particularly relevant for trait regulation in complex plant genomes (Lowder et al., 2015). Recently developed “mini” CRISPRa/i systems based on compact dCas12 proteins (~500 amino acids) further expand these capabilities by allowing multiplexed targeting with reduced interference at chromatin sites, while maintaining transcriptional efficacy across multiple loci (Xu et al., 2021; Pan et al., 2021; Weiss et al., 2022).

These evolving CRISPR-based epigenome editing technologies are powerful tools for investigating epigenetic regulation and enhancing traits in bioenergy crops, with applications that include:

- Functional interrogation of the roles of epigenetic modifications in controlling relevant bioenergy crop traits.
- Establish causal links between epigenetic marks and gene expression by precisely targeting chromatin regulators to specific loci using CRISPR–dCas9 fusion systems.
- Create or modulate stable, heritable epialleles enabling the development of non-GMO phenotypic variants for breeding programs.
- Modulate expression of trait-associated genes in a tunable and reversible manner, allowing precise control of complex polygenic traits in bioenergy crops.
- Engineer gene regulatory networks by multiplexing CRISPR effectors, enabling coordinated reprogramming of epigenetic states across multiple loci.
- Target allele-specific expression in polyploid species to overcome genomic redundancy and achieve precise dosage regulation of key genes.
- Validate candidate regulatory elements identified through single-cell or bulk epigenomic profiling.
- Integrate epigenome editing into epi-breeding pipelines, accelerating the development of resilient, high-yielding bioenergy cultivars.

## 5 Considerations and limitations

While potentially transformative, epigenetic regulation is underexplored and an underutilized avenue for improving traits essential to bioenergy crop productivity. This is likely due to several biological and methodological challenges that continue to limit the practical application of epigenetics in crop development. First, the context-dependent nature of epigenetic marks, shaped by tissue type, developmental stage, and environmental conditions, complicates the identification of reliable, heritable biomarkers for breeding (Tonosaki et al., 2022). While the plasticity of the epigenome is advantageous for stress adaptation, this same variability can hinder trait fixation across generations, making it difficult to distinguish stable epigenetic inheritance from transient responses (Jaligot, 2015). While epigenetic marks can be reversible and context-specific, many are stably inherited across generations through transgenerational epigenetic inheritance (TEI), a distinct subdiscipline of epigenetics with growing significance in breeding strategies (Quadrana and Colot, 2016).

Traditional Mendelian frameworks, which underpin most breeding strategies, often overlook non-Mendelian inheritance patterns characteristic of epigenetic regulation. Mechanisms such as paramutation, transgenerational inheritance, or other epigenetically regulated processes, are likely critical for enhancing desirable traits and therefore can be readily exploited for bioenergy purposes (Tonosaki et al., 2022). Moreover, reliance on the modern models that integrate Mendelian genetics with Darwinian selection, may undervalue the shorter-lived but highly influential epigenetic changes. Even transient modifications persisting only a few generations can significantly enhance bioenergy crop traits, such as growth, yield, and stress tolerance, and offer practical benefits of epigenetically focused performance improvements regardless of long-term evolutionary stability (further discussed by Pembrey, 2015; Tonosaki et al., 2022).

While the exploration of epigenetic regulation as a source of phenotypic variation for bioenergy crop improvement is underway, progress is often constrained by a lack of high-quality, well-annotated reference genomes for many bioenergy crops particularly polyploids and non-model species (Sun et al., 2022). As a result, most mechanistic insights still derive from *Arabidopsis* or major food crops, limiting their immediate transferability to under-characterized bioenergy species. To address this gap, future studies should prioritize generating haplotype-resolved genomic and epigenomic maps under baseline conditions to support accurate genome annotation and functional interpretation. Advances in long-read sequencing platforms now make it feasible to integrate DNA methylation, chromatin structure, and gene regulation across complex plant genomes, but also enable haplotype-resolved epigenomes, which are critical for dissecting allele-specific expression and dosage-dependent regulatory responses (Zhao and Shi, 2025). Building this foundational knowledge will allow for more precise identification of functional epigenetic modifications and regulatory loci, ultimately supporting targeted selection or genome editing strategies to improve energy crop resilience and productivity.

As the global shift toward a bio-based economy accelerates with goals of enhancing energy and food security and developing innovation in sustainable industries, the demand for novel strategies to facilitate this transition grows. Epigenetics, a rapidly evolving field, presents promising opportunities to enhance the breeding and cultivation of bioenergy crops, optimizing both productivity and environmental adaptability, and thereby advancing a more sustainable and resilient bioenergy sector.
